# *Fusarium* Mycotoxins, Their Metabolites (Free, Emerging, and Masked), Food Safety Concerns, and Health Impacts

**DOI:** 10.3390/ijerph182211741

**Published:** 2021-11-09

**Authors:** Theodora I. Ekwomadu, Stephen A. Akinola, Mulunda Mwanza

**Affiliations:** 1 Department of Animal Health, Faculty of Natural and Agriculture, Sciences, Northwest University, Private Bag X2046, Mmabatho 2735, South Africa; akinolastephen3@gmail.com (S.A.A.); mulunda.mwanza@nwu.ac.za (M.M.); 2 Food Security and Food Safety Niche Area, Northwest University, Private Bag X2046, Mmabatho 2735, South Africa

**Keywords:** *Fusarium*, fusariotoxins, mycotoxins, free, emerging, masked, food safety, health impacts

## Abstract

The genus *Fusarium* produces a number of mycotoxins of diverse chemical structures. Fusariotoxins are secondary metabolites produced by toxigenic fungi of the genus *Fusarium*. The important and commonly encountered fusariotoxins are trichothecenes, fumonisins, and zearalenone. *Fusarium* mycotoxins pose varying toxicities to humans and/or animals after consumption of contaminated grain. They can cause acute or chronic illness and, in some cases, death. For instance, a range of *Fusarium* mycotoxins can alter different intestinal defense mechanisms, such as the epithelial integrity, cell proliferation, mucus layer, immunoglobulins, and cytokine production. Of recent concern is the occurrence of emerging and masked *Fusarium* mycotoxins in agricultural commodities, which may contribute to toxic health effects, although the metabolic fate of masked mycotoxins still remains a matter of scientific discussion. These mycotoxins have attracted attention worldwide because of their impact on human and animal health, animal productivity, and the associated economic losses. In this paper, we review *Fusarium* mycotoxins and their metabolites with the aim of summarizing the baseline information on the types, occurrence, and health impacts of these mycotoxins in order to encourage much-needed research on integrated management of this unavoidable food contaminant as concerns for food safety continues to grow worldwide.

## 1. Introduction

Food safety concerns has continued to grow worldwide, including the issue of mycotoxin contamination of food products from farm to fork. Mycotoxin-producing fungi are very common worldwide, occurring in varying amounts in agricultural commodities. These filamentous fungi often grow on edible plants, thus contaminating food and feed with mycotoxins in toxicologically relevant concentrations [1]. Mycotoxin contamination caused by fungal development usually results in a highly concentrated, localized, and inhomogeneous distribution that can spoil an entire grain batch [2]. The presence of mycotoxins in food is almost unavoidable and depends strongly on climatic conditions, and their control is difficult or even impossible. Hence, they present significant hazards to human and animal health. Mycotoxins can adversely affect human and animal health, productivity, economics, and trade [3,4].

The most toxic and prevalent *Fusarium* toxins of economic importance include trichothecenes, fumonisins, and zearalenone. Their importance is attributed to some base-line scientific data as well as significant impact on human health and animal production. These mycotoxins have also been linked to human and animal diseases ranging from acute to chronic and have carcinogenic, mutagenic, teratogenic, estrogenic, hemorrhagic, neurotoxic, hepatotoxic, and immunosuppressive effects [5]. In addition to their significant harmful impacts on health, mycotoxins are major food contaminants affecting global food security, especially in developing countries. The Food and Agriculture Organization (FAO) of the United Nations made an estimate that there was significant contamination of about 25% of the world’s food crops with mycotoxins, leading to annual loss in the range of one million tons [6]. Recent studies suggest that the percentage of contaminated cereals is much higher at 72% [7].

Furthermore, the emergence and occurrence of new *Fusarium* metabolites in food crops and products are of great concern. Emerging mycotoxins produced by *Fusarium* spp., such as fusaproliferin, enniatins, beauvericin, moniliformin, etc., have been reported in food crops, which represents a significant problem in some parts of the world [8,9,10]. The risk to humans and animals from exposure to these mycotoxins has led to persistent elucidation of chemical structures of *Fusarium* mycotoxins in crops and food products. Earlier, scientists observed that some mycotoxicosis symptoms in animals did not correlate with the low mycotoxin content determined in the corresponding feed [11]. The unexpected high toxicity has been attributed to undetected conjugated/masked forms of mycotoxins that were possibly hydrolyzed into the parent toxins in the digestive tract of animals. Accurate risk assessment of masked mycotoxins in foodstuff is difficult owing to the absence of contamination data as well as toxicological properties. Recognition of the toxicological consequence of masked mycotoxins as well as evaluation of the hazard posed by co-occurrence of target mycotoxins contaminating food products has created a new major problem. This should be addressed by food risk assessment and monitoring bodies, food producers, etc. so as to safeguard consumer health and evaluate the health hazards posed by these mycotoxins [12,13].

## 2. Fusarium Mycotoxin Production and Toxicities

*Fusarium* species produce three important classes of mycotoxins, namely trichothecenes, fumonisins, and zearalenones with their mycoestrogens. These toxins are highly toxic and carcinogenic to farm and laboratory animals and have been associated with human esophageal cancer and birth defects [14,15].

### 2.1. Trichothecenes

**Figure 1:**
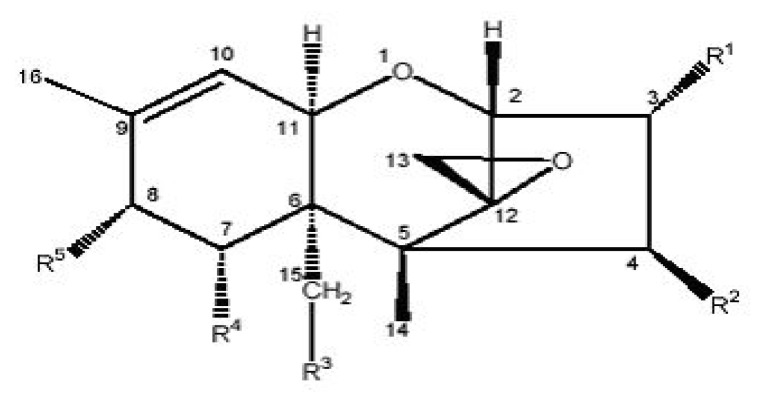
Basic structure of trichothecenes and their respective structures.

Trichothecenes are a very large family (more than 200 different types known presently) of structurally related fungal secondary metabolites produced mainly, but not exclusively, by *Fusarium* species [16,17]. They are a family of naturally occurring tetracyclic sesquiterpenoids and part of a class of terpenes consisting of three isoprene units. Trichothecenes share a common core structure consisting of an olfenic group, an epoxide group, and varying numbers of hydroxyl and acetyl groups (Figure 1 and Table 1). Depending on their functional groups, they can further be classified into one of four groups (A to D), of which groups A and B are the most toxic [18,19] and of the most importance in the context of food [20]. Type A trichothecenes mainly include the highly toxic T-2 toxin (T-2), its deacetylated form HT-2 toxin (HT-2), diacetoxyscirpenol (DAS), and neosolaniol (NEO) [21,22]. Type B trichothecenes, which include deoxynivalenol (DON), nivalenol (NIV), their acetylated derivatives 3-acetyldeoxynivalenol (3-ADON) and 15-acetyldeoxynivalenol (15-ADON), and fusarenon-X (FUS-X), are of great concern for cereal-growing regions worldwide [23]. Trichothecenes generally are of global concern as they are found in cereals usually consumed by livestock and humans, such as maize, barley, oats, and wheat [24,25]. They are potent inhibitors of eukaryotic protein synthesis [26], interfering with initiation, elongation, and termination stages [1]. Some of the diseases associated with these toxins in humans and animals include feed refusal, nausea, vomiting, abortions, weight loss, inflammation of the skin, hemorrhaging of internal organs, blood disorders, immunosuppression, and disturbance of the nervous system [1,20,27].

Emesis, reduced weight gain, and other gastrointestinal disorders are the most sensitive functional manifestations of type B trichothecenes, while immunotoxicity, cytotoxicity, and neurotoxicity are caused by type A trichothecenes [28]. In particular, the intestinal epithelium is the first target tissue of food contaminants, so clinical gastrointestinal outcomes are observed in most cases [29]. Trichothecenes have been shown to be toxic to all species. However, the sensitivity varies considerably between toxins and species, with swine being the most sensitive farm animal. Some of the commonly occurring trichothecenes of *Fusarium* origin are noted below.

### 2.2. Deoxynivalenol

Deoxynivalenol (DON), also referred to as vomitoxin, is produced mainly by *Fusarium graminearum* or by *Fusarium culmorum* in some geographic locations [30]. Deoxynivalenol might co-occur with zearalenone as both toxins can be produced by the same *Fusarium* species. DON mainly affects small grains, such as oats, wheat, and barley, but can also occur in maize [26]. Deoxynivalenol has been designated Group III, meaning they are not classifiable with regard to their carcinogenicity on humans [31]. In dairy cattle, it has been linked to reduced milk. In addition, it causes vomiting in swine consuming contaminated feed or feed refusal because the feed is unpalatable. Reduction in feed intake may lead to serious weight loss, which may impede reproductive performance as well as cause immune system disorders in various animal species [32,33].

Humans are believed to display similar vomiting symptoms when they consume grains that are contaminated with DON [30]. Furthermore, increased prevalence of infections of the upper respiratory tract has been described in children who ingested DON-contaminated wheat bread for over a week. The illness reduced when ingestion of the bread stopped [34]. Deoxynivalenol’s toxicity is assumed to occur through modulation of the innate immune system [35]. Chronic exposure to low dosage of DON may result in anorexia, reduced weight gain, and fluctuation in the production of growth hormone and IgA, while acute exposure to high dosage may induce gastroenteritis, emesis, and a shock-like condition [35]. Deoxynivalenol is unlikely to appear as residues in the tissues or fluids of animals exposed to toxic levels, but baking and malting using DON-contaminated wheat and barley can have adverse effects [30].

### 2.3. Nivalenol

Nivalenol are trichothescence mycotoxins produced by the *Fusarium* fungi, and they can cause problems to pig farmers. Nivalenol-contaminated feed causes feed refusal as the feed is not palatable and can result in vomiting [32]. Maize rations for pigs should not contain excess of 5% contaminated kernels. Cattle can resist some effects from nivalenol contamination, but they do not appear to affect chickens. There have been no reports published so far on the effects of nivalenol on humans. Nivalenol is designated Group III, meaning they are not classifiable with regard to the carcinogenicity on humans [31].

### 2.4. T-2 Toxins

T-2 toxins can cause irritation, hemorrhage, and also necrosis all the way through the gastrointestinal tract (GIT). T-2 toxins also delay cell regeneration of the bone marrow and spleen, weaken immune system functions, and cause changes in the reproductive organs. Infected animals demonstrate signs of weight loss, poor feed utilization, loss of appetite, vomiting, bloodstained diarrhea, abortion, and in severe cases may lead to death [32].

T-2 toxin was the cause of alimentary toxic aleukia (ATA) that killed thousands of people in Russia. Symptoms of alimentary toxic aleukia include fever, nose bleeding, and also bleeding from the throat, skin, and gums. It also causes necrosis and other than the cytotoxic effects, suppresses the immune system [1].

### 2.5. Fumonisins

**Figure 2:**
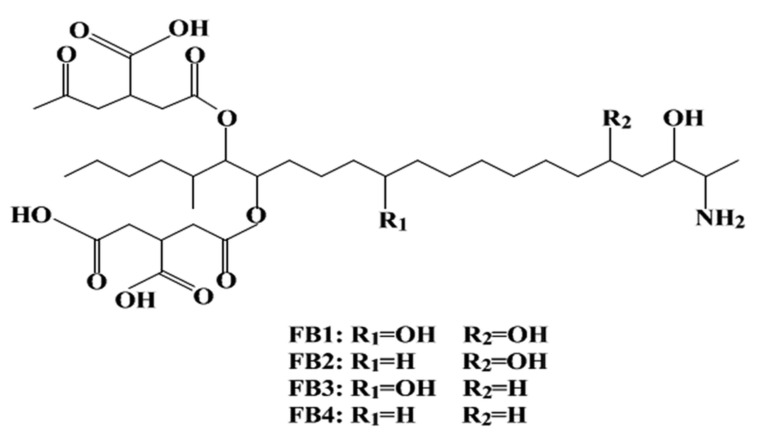
Chemical structure of fumonisin B.

Fumonisins were firstly discovered in 1988, and they are some of the most cytotoxic and carcinogenic mycotoxins [36]. Fumonisin analogues are divided into four types, namely fumonisin A, B, C, and P series. To date, 28 structurally related fumonisin analogues have been described. Fumonisin B (Figure 2) is the most important and naturally widespread fumonisin in contaminated maize. Fumonisin B analogues comprise toxicologically significant fumonisin B1, B2, and B3. These fumonisin Bs occur naturally in abundance, with fumonisin B1 dominating with the highest concentration [37]. They are produced by *Fusarium verticillioides*, which was formerly known as *Fusarium moniliforme*, a common fungal pathogen of maize. *Aspergillus niger*, on the other hand, has now been found to produce fumonisin B2 (FB2) [38]. Fumonisin B1 has been implicated in human esophageal cancer in the Transkei region of South Africa. This is associated with the ingestion of fumonisin-contaminated maize. Similar observations have been made in China and Northeast Italy [1,39]. As stated by Marasas et al. [40], because FB1 is capable of reducing folate absorption in different cell lines, it is likely to be connected with neural tube defects in infants. Fumonisin B1 has also been associated with high incidence of neural tube defects in babies of mothers who consumed fumonisin-contaminated maize near the Texas–Mexico boundary [41]. Chronic intake of fumonisin mycotoxins has also been associated with impaired growth in children [42]. The IARC designated FB1 in Group II B as “possibly carcinogenic to humans” [43]. Fumonisin B1 structurally resembles sphingoid bases, which can explain why the biosynthesis of sphingolipid complexes is inhibited and leads to cell destruction and ensuing cell death [30,44]. Fumonisins are somewhat heat stable; however, in heat-processed foods, FB1 exists in covalently bound forms [30]. Unlike other very well-known mycotoxins that can dissolve in organic solvents, fumonisins are water soluble, which makes them a challenge to study [1]

Although fumonisins have a somewhat simple chemical structure, their blockage of sphingolipid metabolism can have diverse and complex effects on various animal systems [20] Fumonisins damage functions of the immune system, liver, and kidney; cause weight reductions; and increase mortality rates. The most sensitive animals to fumonisin toxicity are horses, and levels at the range of 0.2–126 ppm FB1 were found in feed samples associated with outbreaks of leukoencephalomalacia (LEM) (hole in the head syndrome), which is a necrosis of the brain in North America, South America, and South Africa [45,46]. Porcine pulmonary oedema is associated with the consumption of fumonisin-contaminated feed [47,48]. It has also been shown that fumonisin B1 is hepatocarcinogenic to rats. Susceptibility to fumonisins may be higher in dairy cattle than in beef cattle due to major production stress [49].

### 2.6. Zearalenone and Its Metabolites (Mycoestrogens)

**Figure 3:**
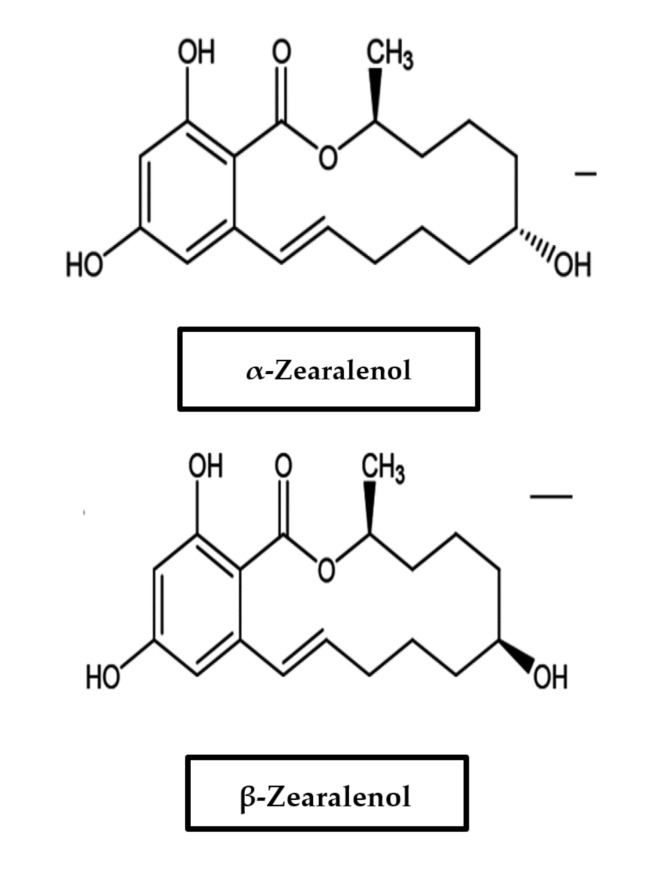

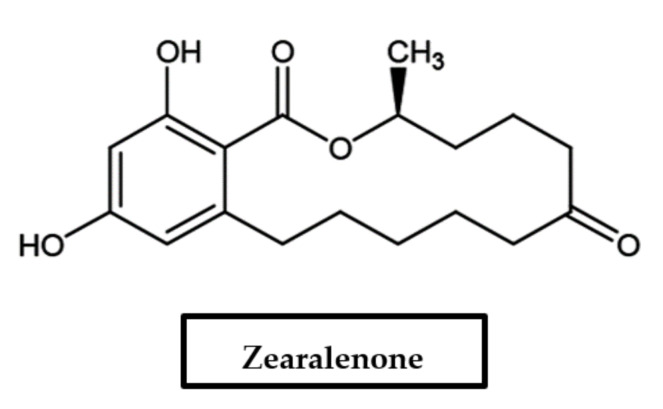
Chemical structure of zearalenone, α-zearalenol, and β-zearalenol.

Zearalenone (ZON) and its metabolites are termed mycoestrogens, a subgroup of naturally occurring estrogenic compounds. Being the principal representative of this group of nonsteroidal mycoestrogens, zearalenone is a 6-(10-hydroxy-6-oxo-trans-1-undecenyl)-β-resorcylic acid lactone (Figure 3). It is biosynthesized through a polyketide pathway. The keto group at C-8 is reduced to α- and β-isomers in mammalian tissues, and these metabolites can be produced at low concentrations by the fungi. Production by *F. graminearum, F. crookwellense, F. sporotrichioides*, and *F. culmorum* has been mainly described, and co-occurrence is contingent on DON and other trichothecenes [50]. In addition, following oral exposure, zearalenone is metabolized in diverse tissues, predominantly in the liver, with α-zearalenol and β-zearalenol being the main metabolites [51].

Zearalenone, also known as F-2, commonly contaminates maize but can also occur in other crops worldwide [26,30,52]. Several authors have reported on the occurrence of ZON in foods as well as in animal feeds from various African countries, such as South Africa [53] and Nigeria [54]. Being classified as endocrine disruptor chemicals, they are usually suspected of reducing fertility in males in human and wildlife populations and possibly involved in the development of numerous types of cancers [55]. There have been claims that the high rate of premature menarche in Puerto Rico could be a result of ZON and related compounds in the diet [1]. Due to its high estrogenic action, zearalenone has been used for the treatment of postmenopausal symptoms in women. Despite that, research has revealed the potential for ZON to aggravate the development of human breast cancer cells with estrogen response receptors [56]. While the biological potency of this compound is high, its real toxicity is low [1]. It has been placed in Group III, meaning not classifiable with regard to their carcinogenicity to humans [31]. Because ZON seems to bind to estrogen receptors [30], it has been implicated in a number of mycotoxicoses in farm animals. The consumption of contaminated grains by farm animals can lead to the manifestation of female features in males, early sexual development of young females, infertility in adults, abortion, false heat, recycling, stillbirth, birth of malformed offspring, reabsorption of fetuses, and mummies. It can also cause atrophy of the testes and weakened libido in males. Cattle might have reddened, inflamed, or swollen vulvas and enlarged nipples, and vaginal and rectal prolapse may possibly follow. It also causes reduced feed intake and possibly feed refusal. Its effects are most noticeably seen in pigs; in fact, pigs seem to be the most susceptible species to ZON [30,52]. The recommended safe daily human consumption of ZON is estimated to be 0.05 microgram per kilogram body weight [1]. Less common or emerging *Fusarium* mycotoxins include moniliformin, fusarin C, enniantins, beauvericin, and fusaproliferin (see Section 3).

## 3. Emerging Fusarium Toxins

“Emerging toxins” are a group of chemically different mycotoxins for which no regulations exist at present. Current studies using cutting-edge LC–MS/MS for structure elucidation offer insights regarding newly discovered metabolites, as do plant breeding efforts adjusting to climate change [57]. Risk assessment studies are ongoing in preparation for legislation when considered conclusive [58]. Normally referred to in this group are moniliformin (MON), fusarin C, beauvericin (BEA), enniantins (ENNs), and fusaproliferin (FUS).

### 3.1. Moniliformin

Moniliformin, which has the chemical formula 3-hydroxycyclobut-3-ene-1,2-dione, is an organic acid that exists as sodium or potassium salt in nature. It is therefore extremely water soluble due to its high polarity [59]. Moniliformin is known to be a worldwide natural contaminant in cereals such as rice, wheat, oats, barley, rye, and maize [60]. It is produced by different species of *Fusarium* but mostly by *Fusarium proliferatum* [61]. Its natural occurrence was firstly reported in 1982 in moldy maize obtained from the Transkei region of South Africa at levels ranging from 16 to 25 ppm [62]. Moniliformin usually co-occurs with other *Fusarium* mycotoxins, such as FB1, ZON, trichothescenes, fusarin C, and beauvericin. In humans, MON consumption has been associated with some disease outbreaks, for example, the case of Keshan disease, which is a myocardia disease that occurred in the rural areas of China and South Africa (Table 2), [63].

### 3.2. Enniatins

Structurally, ENNs are cyclohexadepsipeptides composed of alternating residues of three N-methyl amino acids and three hydroxyl acids. They are lipophilic in nature, which allows them to be integrated into the lipid bilayers of cell membranes [64]. Enniatins have antimicrobial, insecticidal, and antifungal properties and may also have herbicidal properties. Their mechanism of action is in the direction of cellular membrane transport proteins, which are inhibited by the toxin. The toxicity of enniatins is in particular profound towards mitochondria. These organelles are vital constituents of living beings and are responsible for respiration in the cell, producing most of the adenosine triphosphate (ATP) necessary for energy transfer. Enniatins do seem to be efficiently degraded in animal intestinal systems, but more research is necessary [65].

### 3.3. Beauvericin

Beauvericin (BEA) is a cyclic hexadepsipeptide that is synthesized by various toxigenic fungi. It usually inhibits cholesterol acyltransferase [66]. It shows strong antimicrobial activity towards a broad spectrum of bacteria, with no distinction between Gram-positive and Gram-negative bacteria. This toxin also shows cytotoxic, apoptotic, and immunosuppressive activity. Beauvericin acts on the cellular membranes, increasing permeability and disrupting the cellular homeostasis. Although moniliformin shows a relatively lower toxicity compared to enniatins and beauvericin, it has been reported to be toxic towards lymphocytes, skeletomyocytes, and cardiomyocytes, with birds and minks being the most sensitive species. The mechanism of action has not yet been fully elucidated, but toxicity towards mitochondria with a mechanism similar to enniatins is suspected [65].

### 3.4. Fusaproliferin

Fusaproliferin (FUS) is a bicyclic sesterterpene consisting of five isoprenic units, identified from maize cultures of *F. proliferatum*. Fusaproliferin has demonstrated toxicity towards human B-lymphocytes and some insect cell lines [59]. This emerging mycotoxin also showed teratogenic and pathogenic effects on chicken embryos. In recent years, some studies have been conducted using brine shrimp (*Artemia salina*) as a model organism, and FUS was found to be toxic to *Artemia salina*. The toxin often co-occurs with deacetylfusaproliferin, although the toxicity of the latter is much lower compared to fusaproliferin [65].

Studies on the synergistic effects between the two toxins have not been reported so far [65].

## 4. Masked Mycotoxins

### 4.1. Introduction

In the past few decades, it has become clear that many structurally related compounds generated by plant metabolism, fungi, or food processing coexist with their parent mycotoxins in mycotoxin-contaminated commodities. In the mid-1980s, the topic of masked mycotoxins received attention because clinical observations in animals in some cases of mycotoxicosis did not correlate with the low mycotoxin content determined in the corresponding feed [11]. The unexpected high toxicity has been attributed to undetected conjugated forms of mycotoxins that were possibly hydrolyzed into the parent toxins in the digestive tract of animals.

Young et al. [81] showed that the DON content of yeast-fermented food products was higher than that of the contaminated flour used for their production. Therefore, it has been speculated that there might exist a DON conjugate of some form arising from plant metabolism. Savard [82] was the first to chemically synthesize glucose and fatty-acid conjugates of DON, while Sewald et al. [83] could identify deoxynivalenol-3-glucoside (DON-3G) as a DON metabolite in maize cell suspension cultures. The glycosylated form of ZON, zearalenone-14-glucoside (ZON-14G), is certainly the best studied conjugated form. It was originally found as a fungal metabolite [84], but Engelhardt et al. [85] reported that ZON was also transformed to ZON-14G by plant cultures. Gareis et al. [11] hypothesized that ZON-14G was cleft during digestion in swine, releasing the estrogenic aglucone ZON. Sulfate and glucuronide conjugates of ZON were shown to occur in urine of ZON-fed animals [86], although their identity could not be confirmed. Zearalenone-14-sulfate (ZON-14S) was isolated by Plasencia and Mirocha [87] as a fungal metabolite from *Fusarium*.

Two types of masked mycotoxins can be distinguished, namely extractable conjugated and nonextractable bound varieties. Extractable conjugated mycotoxins can be detected by appropriate analytical methods when their structure is known and analytical standards are available. Nonextractable bound mycotoxins are covalently or noncovalently attached to polymeric carbohydrate or protein matrices. Bound mycotoxins are not directly accessible and have to be liberated from the matrix by chemical or enzymatic treatment prior to chemical analysis.

### 4.2. Formation and Occurrence of Masked Mycotoxins

Mycotoxins can be subjected to biological modification through conjugation by plants or through chemical modification, either thermally or nonthermally, e.g., by food processing. As indicated earlier, these modified mycotoxins can contribute to the degree of contamination and may escape detection methods, causing an underestimation of the mycotoxin exposure and risk [88]. The different mechanisms are noted below.

#### 4.2.1. Plant Conjugates

It has been shown that analogously to animals, plants as living organisms have the capability to defend themselves against the potentially toxic effects of xenobiotic compounds, including mycotoxins. The defense mechanisms of plants include three phases of biosynthetic pathways. Phase I is the transformation or activation phase, phase II is the solubilization or conjugation phase, and phase III is known as the compartmentalization phase [88,89].

After the infection of crop plants, mycotoxins are modified or transformed by introducing reactive groups into the toxic molecules using plant enzyme (esterases, amidases, and the cytochrome P-450 system). The biotransformed molecules are then conjugated to more polar substances (often with sugars, amino acids, or sulfates) with the help of plant enzymes, i.e., glucosyltransferases attaching a glucose molecule to the toxin and increasing its water solubility. Conjugated forms are compartmentalized in specific organelles (e.g., vacuoles and chloroplasts) or in extracytoplasmic space (e.g., cell wall) of plant cells [90,91,92,93]. Although these conjugated mycotoxins are unable to exert harmful effects to the plant because they have been compartmentalized, the residues may persist for considerable periods of time in plants and can have important toxicological consequences for consumers.

**Figure 4:**
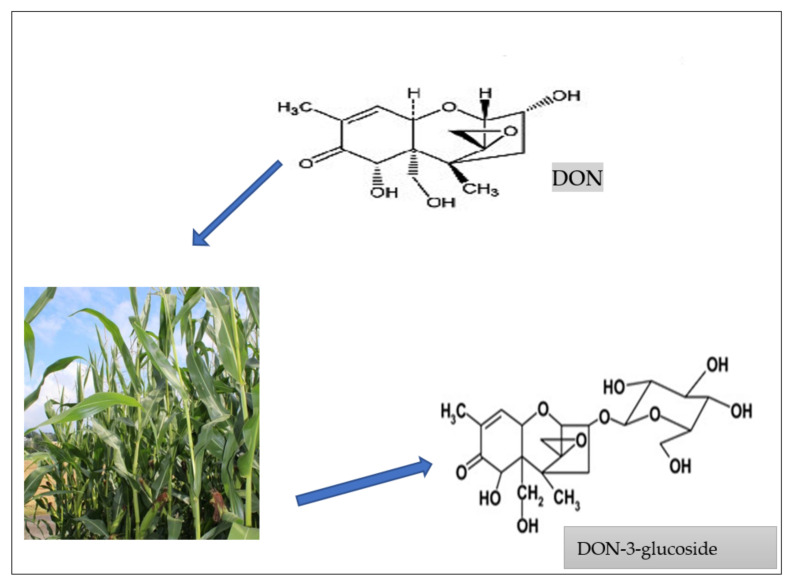
How plants metabolize free mycotoxins to form masked mycotoxins, e.g., DON forms DON-3-glucoside [97,99].

In planta transformation of mycotoxins has been predominantly described for *Fusarium* toxins [94]. Because *Fusarium* infection normally occurs in the field, *Fusarium* mycotoxins, e.g., deoxynivalenol (DON), zearalenone (ZON), fumonisins (FUM), nivalenol (NIV), fusarenon-X (FUS-X), T-2 toxin, HT-2 toxin, and fusaric acid, are the biggest targets for conjugation [95]. Metabolism by the host plant is generally aimed at detoxification of these compounds, which is often accomplished by attachment of hydrophilic groups, thus increasing their solubility in water [92,96]. In this manner, for example, DON is converted to DON-3-glucoside (D3G) [83,97] (Figure 4), while ZON can be altered to ZON-4-glucoside (Z4G) [85] by plants. Moreover, Nakagawa et al. [98] reported glucoside forms of other trichothecenes, such as nivalenol (NIV) in artificially contaminated wheat.

#### 4.2.2. Food Processing Conjugates

Besides plant metabolism, processing of food commodities is another source of mycotoxin conjugate formation. The (thermo) stability of *Fusarium* mycotoxin trichothecenes allows them to withstand most food and feed processes [100]. The technological process also has an important role in the masking process, particularly in cereal-derived products. Indeed, mechanical or thermal energy during the transformation process may cause significant modification, for instance, the induction of reactions with macromolecular components, such as polysaccharides, proteins, or lipids, or the release of native toxins through decomposition of the masked derivatives. In the case of fumonisins, the phenomenon has been described as “the fumonisin paradox” because low contaminated commodities have apparently also been found to induce toxic effects. This highlights the problem of “bound” or “hidden” fumonisins, which may be released upon alkaline hydrolysis [101,102]. The effect of food being thermally processed on chemical structure as well as on the toxicity of fumonisins has been extensively reviewed [103]. A major reaction occurring in heat-treated food involves fumonisin B1 (FB1) and reduction of sugars to form N-carboxymethyl-FB1 [104] and N-1-deoxy-D-fructos-1-yl-FB1 [105]. The occurrence of these fumonisin derivatives has been described in corn products [106]. On the other hand, low recoveries of FB1 have been observed in different matrices, such as rice flour, cornstarch, cornmeal, and glucose after thermal treatment [107]. It is believed that FB1 interacts with food macroconstituents, such as protein or starch. In order to yield evidence that FB1 might bind to matrix components in thermally treated food, model experiments were performed [108,109,110]. The results indicated that fumonisins could bind to polysaccharides and proteins via their two tricarboxylic acid groups and that binding occurred to a greater extent in starch than in proteins. The occurrence of protein-bound fumonisins in commercial corn flakes has also been shown [108,109,111].

Similarly, microorganisms used in the fermentation process (introduction of yeast) or malting process may transform mycotoxins into products that are also not detected by analytical methods. These derivatives are formed from enzymatic activities of microbial cultures used for fermentation, such as in manufacturing beer, wine, fermented sausages, or mixed pickles. Another food commodity that is frequently contaminated with modified mycotoxins is bread. During the processing of wheat to bread, it has been described that milling had little influence on the ratio of DON3G to DON. Due to fractionation, milling decreased the DON3G and DON content in white flour compared to initial unprocessed wheat [112]. These findings are supported by a previous study in which the fractionation of *Fusarium* mycotoxins during dry milling of maize was investigated. It was observed that bran, the hard outer layers of cereal grain that are discarded for the production of white flour, contained the highest concentrations of all tested mycotoxins [113]. During kneading, fermenting, and proofing, no significant changes occurred for DON, DON3G, and 3ADON. However, when bakery improvers, such as enzyme mixtures, were employed as a dough ingredient, a distinct increase in DON3G was noticed in fermented dough [112,114]. It is assumed that this increase in DON3G is due to release from starch-based, matrix-bounded forms. The effect of food processing on T2-G, HT2-G, and FUS-X has also been investigated, although to a lesser extent [114,115]. Data on the fate of α-ZOL, β-ZOL, ZON14G, α-ZOL4G, β-ZOL4G, and ZON14 during food processing are also available [88].

## 5. Health Effects of Masked Mycotoxins Using Studies on Human Cell Lines, Animals, and Plants

### 5.1. Zearalenone and Its Modified Derivatives

The toxicity of the zearalenone-modified forms α- and β-ZOL has been widely described, with the majority of the studies focusing on estrogenicity and reproductive disorders. Despite the pronounced estrogenic effects of ZON and its metabolites, there have also been reports of their cytotoxicity. For example, a study was carried out on cell viability using Caco-2 cell lines in MTT assay after 48 h incubation with ZON, α-ZOL, or β-ZOL as well as oxidative stress induction by measuring malondialdehyde generation. The results showed that both metabolites demonstrated decreased toxicity compared to ZON, with the following ranking: α-ZOL < β-ZOL < ZON. The measurement of DNA lesions due to oxidative stress also resulted in a similar relative ranking of toxicity [116].

### 5.2. Deoxynivalenol (DON) and Its Modified Derivatives

The toxicity of DON is mainly due to the inhibition of protein synthesis. It is known to cause irritation of the gastrointestinal (GI) tract. In fact, it can be argued that most mycotoxins do not cross the intestinal barrier, so not all mycotoxins will cause mycotoxicoses. Still, the first cells to be exposed to mycotoxins are the gastrointestinal cells, and they are exposed at greater concentrations than in other tissues. Therefore, unabsorbed toxins can affect the intestinal epithelium and its whole extension before absorption can even begin. Hence, a provisional tolerable dietary intake (TDI) for DON has been set at 1 μg/kg body weight [117].

Several studies have been carried out to investigate the toxicity of DON and its modified derivatives using human cell lines. For example, Alassane-Kpembi et al. [118] investigated the influence of DON, 3ADON, and15ADON on human epithelial colorectal adenocarcinoma cell line (Caco-2), and the induced cytotoxicity was measured by means of MTT assay. The results showed that 15ADON was more toxic than DON and 3ADON as it exhibited higher inhibition of epithelial cell viability. Another study by Kadota et al. [119] compared the toxicity of these compounds by measuring their effect on interleukin-8 (IL-8) secretion and intestinal transport in Caco-2 cells. A significantly higher absorption of 15ADON compared to DON and 3ADON was demonstrated, and 15ADON had a more profound effect on intestinal integrity, facilitating absorption through passive diffusion. Furthermore, the production of IL-8 was the lowest after 3ADON exposure, followed by DON and 15ADON [119,120]. Generally, the majority of the modified mycotoxin toxicity research has been dedicated to human in vitro toxicity, while less research has been carried out using animals and plants.

## 6. Management of Food Safety Risks of *Fusarium* Mycotoxins

For years now, scientists have proposed different ways of mitigating food safety risks with regard to fungal and mycotoxin contamination of agricultural commodities in the food system. Implementation of good agricultural practices (GAP), good manufacturing practices (GMP), and hazard analysis and critical control points (HACCP), amongst other measures, have been proposed. The adoption of GAP in the field and during storage, GMP during food processing, and HACCP during food handling and packaging throughout the whole field-to-table chain will go a long way to address the problems caused by fungi and mycotoxins in the food system [121,122].

(a) Appropriate agronomic practices include the use of resistant crop varieties that can resist insect and fungal infection of crops in the field. Crop rotation, e.g., maize–soy bean rotation, reportedly results in less outbreaks of *Fusarium* than maize–maize planting operations [123]. Proper and uniform irrigation helps to reduce plant drought stress, which has been reported to favor mycotoxin (fumonisin) contamination [15]. Getting rid of grasses, weeds, debris, alternate host plants, crop residues, etc., which can serve as reservoirs to fungal inoculums, can also prevent mycotoxin contamination in the field. Moreover, reducing mechanical damage to crops to a minimum during harvesting reduces mycotoxin contamination as mycotoxins are generally higher in broken kernels. Appropriate and approved fungicides, pesticides, and other chemicals also prevent mycotoxin contamination in grains, although they should be used with caution as their residues could be toxic to mammalian cells [124]). Drying of food and feed to safe moisture levels during storage will also help prevent mycotoxin formation.
(b) Food processing includes physical techniques, such as cleaning and milling processes, physical adsorption, and thermal processes; chemical techniques, such as the use of ammonia, calcium hydroxide, and sulfur-containing compounds; and biological techniques, such as malting, brewing, and fermentation [121]. Studies have reported the positive effect of physical decontamination methods, such as sorting, washing, dehulling, etc., on reducing mycotoxins in grains [125,126]. Moreover, mycotoxins are thermally stable [127] and tend to survive thermal processing, even when cooked at pretty high temperatures, such as those reached during bread making or breakfast cereal production [128]. Nevertheless, reduction has also been reported at very high temperatures, although this may be due to reactions resulting in the formation of products with altered chemical structures. The effectiveness of thermal treatment (extrusion cooking) on the reduction of some *Fusarium* toxins, e.g., zearalenone at temperatures ranging from 120 to 160 °C, has also been reported [129]. Fermentation, which is a universal biological food processing method, has also been reported to reduce mycotoxin contamination, e.g., a 50% reduction in deoxynivalenol was recorded during traditional beer processing [130], In spite of this, the amount of reduction in mycotoxin contamination in food and feed products by processing is dependent on the matrix type, the mycotoxin, and the processing method and conditions used. Besides studying the effect of food processing on mycotoxins, it is important to be aware of the possibility of free mycotoxins co-occurring with their masked forms or free mycotoxins being modified and fragmented into other forms during food processing, which may not be easily detected by routine analytical methods.
(c) Food handling and packaging includes the use of technologies such as modified atmosphere packaging (MAP), which is a useful technology that involves the use of oxygen (O_2_) absorbents, storage temperature, and packaging film barrier. MAP can be used to prevent fungal growth and mycotoxin contamination on finished products [122,124]

Creating awareness of the importance of adopting GAP, GMP, and HACCP to control (prevention and decontamination) mycotoxin metabolites in the food system will be useful in reducing the risk of mycotoxin exposure to some extent. Mycotoxin control strategies should also include approaches for the diversion of contaminated agricultural products to lower-risk uses.

## 7. Conclusions

Generally, it can be possibly argued that metabolism of *Fusarium* mycotoxins in plants are a detoxification pathway that seemingly results in less toxic or inactive compounds via increased water solubility of the mycotoxins, which facilities their excretion and hence results in decreased toxicity [131]. Contrary to this, most recent studies have shown that most masked mycotoxins are not functionally uniform and that their toxicity greatly depends on the chemical properties of the mycotoxins in question and the exposure [132]. However, further research is necessary to fully clarify their physiological and toxicological roles as some of these toxins may not act as their parent compounds. The outcome of these toxicological studies will hopefully prompt legislators to expand regulations to cover these *Fusarium* masked mycotoxins, which will be immensely important for the integrated management of this unavoidable food contaminant.

Despite the progress in mycotoxin analysis, there are limitations to understanding their impact on animal and human health through food products. Co-contamination by different *Fusarium* mycotoxins and identification of some forms of new ones both necessitate new toxicological studies for assessment. Some regulations, especially those established by the European Union, have gradually recognized the risk of contamination by mycotoxins in the food chain. Although existing in vitro studies have shown that masked forms have lower toxic effects on animal and human cells than free mycotoxins, in vivo studies have demonstrated that masked forms have significant toxicity due to their conversion to the free form by enzymatic reactions. As such, there is a need to constantly and routinely monitor the occurrence of *Fusarium* mycotoxins and their modified forms in food and feed commodities as the annual levels may vary depending on environmental moisture, climate change, temperature differences, plant disease status, insect pest numbers, etc. Effective management of food safety risks is required, especially the use of rapid and sensitive immunological techniques.

## Figures and Tables

**Table 1:** Trichothecenes and their structures.

Trichothecene	R1	R2	R3	R4	R5
		TYPE A			
HT-2 toxin	OH	OH	OAc	H	OCOCH_2_CH(CH_3_)_2_
T-2 toxin	OH	OAc	OAc	H	OCOCH_2_CH(CH_3_)_2_
Diacetoxyscirpentriol	OH	OAc	OAc	H	H
		TYPE B			
Deoxynivalenol	OH	H	OH	OH	O
3-acetyl-deoxynivalenol	OAc	H	OH	OH	O
15-acetyl-deoxynivalenol	OH	H	OAc	OH	O
Nivalenol	OH	OH	OH	OH	O
Fusarenon X	OH	OAc	OH	OH	O

**Table 2:** Health effects of some *Fusarium* mycotoxins.

Mycotoxinss	Health Effects	Organs Affected	References
Fumonisin	Carcinogenic, hepatotoxic, nephrotoxic, and immunosuppressive.	Gastrointestinal tract (GIT), liver, and kidney	Soriano and Dragacci, 2014 [67,68,69]
Deoxynivalenol	Nausea, vomiting, diarrhea, reproductive effects, and toxicosis	Reproductive organs and GIT	Richard,2007 [30,70]
T-2 Toxin	Hepatotoxic, genotoxic, and immune-suppressive	Gastrointestinal tract (GIT) and immune system	Hymery et al., 2009 [71,72]
Nivalenol	Annorexic immunotoxic, haematotoxic, and genotoxic	Gastrointestinal tract (GIT) and muscle	Bony et al., 2007 [73,74]
Zearalenol	Carcinogenic, hormonal imbalance, and reproductive effects	Reproductive organs	D”Mello and Macdonald, 1997 [75,76]
Moniliformin	Cardiotoxic and muscular disorders	Heart, kidney, and muscle	Zang et al., 2007 [77,78]
Enniatins	Immunotoxic and cytotoxic	Immune system	Prosperini et al., 2014 [79,80]

## Data Availability

Not applicable.

## References

[1] Bennett J.W., Klich M. (2003). Mycotoxins. Clin. Microbiol. Rev..

[2] Casado M.R., Parsons D., Weightman R., Magan N., Origgi S. (2009). Modelling a two-dimensional spatial distribution of mycotoxin concentration in bulk commodities to design effective and efficient sample selection strategies. Food Addit. Contam. Part A.

[3] Smith J.E., Solomons G., Lewis C., Anderson J.G. (1995). Role of mycotoxins in human and animal nutrition and health. Nat. Toxins.

[4] Wild C.P., Gong Y.Y. (2010). Mycotoxins and human disease: A largely ignored global health issue. Carcinogenesis.

[5] Yazar S., Omurtag G.Z. (2008). Fumonisins, Trichothecenes and Zearalenone in Cereals. Int. J. Mol. Sci..

[6] Smith J.E., Solomons G., Lewis C., Anderson J.G. (1994). Mycotoxins in Human Nutrition and Health.

[7] Streit E., Naehrer K., Rodrigues I., Schatzmayr G. (2013). Mycotoxin occurrence in feed and feed raw materials worldwide: Long-term analysis with special focus on Europe and Asia. J. Sci. Food Agric..

[8] Kosiak B., Torp M., Skjerve E., Thrane U. (2003). The prevalence and distribution of Fusarium species in Norwegian cereals: A survey. Acta Agric. Scand. Sect. B Plant Soil Sci..

[9] Rokka M., Yli-Mattila T., Parikka P., Rizzo A., Peltonen K. (2004). Presence and concentrations of the Fusarium-related mycotoxins beauvericin, enniatins and moniliformin in finnish grain samples. Food Addit. Contam..

[10] Ekwomadu T.I., Dada T.A., Nleya N., Gopane R., Sulyok M., Mwanza M. (2020). Variation of Fusarium free, masked, and emerging mycotoxin metabolites in maize from agriculture regions of South Africa. Toxins.

[11] Gareis M., Bauer J., Thiem J., Plank G., Grabley S., Gedek B. (1990). Cleavage of zearalenone-glycoside, a “masked” mycotoxin, during digestion in swine. J. Vet. Med. Ser. B.

[12] Stoev S.D., Denev S.A. (2013). Porcine/chicken or human nephropathy as the result of joint mycotoxins interaction. Toxins.

[13] Stoev S.D. (2015). Foodborne mycotoxicoses, risk assessment and underestimated hazard of masked mycotoxins and joint mycotoxin effects or interaction. Environ. Toxicol. Pharmacol..

[14] Leslie J.F., Summerell B.A. (2006). The Fusarium Laboratory Manual.

[15] Ekwomadu T., Mwanza M., Rios C. (2015). A decade of mycotoxin research in Africa: A review. Mycotoxins, Occurrence, Toxicology and Management Strategies.

[16] Zhou T., He J., Gong J. (2008). Microbial transformation of trichothecene mycotoxins. World Mycotoxin J..

[17] Foroud N.A., Baines D., Gagkaeva T.Y., Thakor N., Badea A., Steiner B., Bürstmayr M., Bürstmayr H. (2019). Trichothecenes in cereal grains—An update. Toxins.

[18] Wu Q., Huang L., Liu Z., Yao M., Wang Y., Dai M., Yuan Z. (2011). A comparison of hepatic in vitro metabolism of T-2 toxin in rats, pigs, chickens and carp. Xenobiotica.

[19] Shank R.A., Foroud N.A., Hazendonk P., Eudes F., Blackwell B.A. (2011). Current and future experimental strategies for structural analysis of trichothecene mycotoxins—A prospectus. Toxins.

[20] Desjardins A.E. (2006). Fusarium Mycotoxins: Chemistry, Genetics and Biology.

[21] Thrane U., Adler A., Clasen P.-E., Galvano F., Langseth W., Lew H., Logrieco A.F., Nielsen K.F., Ritieni A. (2004). Diversity in metabolite production by *Fusarium langsethiae*, *Fusarium poae*, and *Fusarium sporotrichioides*. Int. J. Food Microbiol..

[22] Rocha O., Ansari K., Doohan F.M. (2005). Effects of trichothescene mycotoxins on eukaryotic cells: A review. Food Addit. Contam..

[23] Jurado M., Vázquez C., Patiño B., González-Jaén M.T. (2005). PCR detection assays for the trichothecene-producing species *Fusarium graminearum*, *Fusarium culmorum*, *Fusarium poae*, *Fusarium equiseti* and *Fusarium sporotrichioides*. Syst. Appl. Microbiol..

[24] Eriksen G., Petterson H. (2004). Toxicological evaluation of trichothecenes in animal feed. Anim. Feed. Sci. Technol..

[25] Wu Q., Kuca K., Humpf H., Klímová B., Cramer B. (2017). Fate of deoxynivalenol and deoxynivalenol-3-glucoside during cereal-based thermal food processing: A review study. Mycotoxin Res..

[26] Council for Agriculture Science and Technology (CAST) (2003). Mycotoxins: Risks in Plant, Animal, and Human Systems.

[27] Logrieco A., Bottalico A., Mulé G., Moretti A., Perrone G. (2003). Epidemiology of toxigenic fungi and their associated mycotoxins for some Mediterranean crops. Eur. J. Plant. Pathol..

[28] Van der Fels-Klerx H.J., Stratakou I. (2010). T-2 toxin and HT-2 toxin in grain and grain-based commodities in Europe: Occurrence, factors affecting occurrence, co-occurence and toxicological effects. World Mycotoxin J..

[29] Pinton P., Tsybulskyy D., Lucioli J., Laffitte J., Callu P., Lyazhri F., Grosjean F., Bracarense A.P., Kolf-Clauw M., Oswald I. (2012). Toxicity of deoxynivalenol and its acetylated derivatives on the intestine: Differential effects on morphology, barrier function, tight junction proteins, and mitogen-activated protein kinases. Toxicol. Sci..

[30] Richard J.L., Richard J.L. (2000). Mycotoxins—An overview. Romer Labs’ Guide to Mycotoxins.

[31] International Agency for Research on Cancer (IARC) (1993). Some Naturally Occurring Substances. Food Items and Constituents, Heterocyclic Aromatic Amines and Mycotoxins.

[32] Agag B.I. (2005). Mycotoxins in foods and feeds. 5-trichothecenes, A T-2 toxin. Assiut Univ. Bull. Environ. Res..

[33] Pestka J.J., Amuzie C.J. (2008). Tissue distribution and proinflamatory cytokine gene expression following acute oral exposure to deoxynivalenol: Comparison of weaning and adult mice. Food Chem. Toxicol..

[34] Hopton R.P., Turner E., Burley V.J., Turner P.C., Fisher J. (2010). Urine metabolite analysis as a function of DON exposure: An NMR-based metabolomics investigation. Food Addit. Contam. Part. A Chem. Anal. Contr. Expo. Risk Assess..

[35] Pestka J.J. (2010). Deoxynivalenol-induced pro-inflammatory gene expression: Mechanisms and pathological sequelae. Toxins.

[36] Gelderblom W.C., Jaskiewicz K., Marasas W.F., Thiel P.G., Horak R.M., Vleggaar R., Kriek N.P. (1988). Fumonisins—Novel mycotoxins with cancer-promoting activity produced by *Fusarium moniliforme*. Appl. Environ. Microbiol..

[37] Marasas W.F.O., Jackson L.S., DeVries J.W., Bullerman L.B. (1996). Fumonisins: History, worldwide occurrence and impact. Fumonisins in Food.

[38] Frisvad J.C., Smedsgaard J., Samson R.A., Larsen T.O., Thrane U. (2007). Fumonisin B2 production by *Aspergillus niger*. J. Agric. Food Chem..

[39] Peraica M., Radić B., Lucić A., Pavlović M. (1999). Toxic effects of mycotoxins in humans. Bull. World Health Organ..

[40] Marasas W.F.O., Riley R.T., Hendricks K.A., Stevens V.L., Sadler T.W., Gelineau-van Waes J., Missmer S.A., Cabrera J., Torres O., Gelderblom W.C.A. (2004). Fumonisins disrupt sphingolipid metabolism, folate transport, and neural tube development in embryo culture and in vivo: A potential risk factor for human neural tube defects among populations consuming fumonisin-contaminated maize. J. Nutr..

[41] Missmer S.A., Suarez L., Felkner M., Wang E., Merrill A.H., Rothman K.J., Hendricks K.A. (2006). Exposure to Fumonisins and the occurrence of neural tube defects along the Texas–Mexico border. Environ. Health Perspect..

[42] Kimanya M.E., de Meulenaer B., Roberfroid D., Lachat C., Kolsteren P. (2010). Fumonisin exposure through maize in complementary foods is inversely associated with linear growth of infants in Tanzania. Mol. Nutr. Food Res..

[43] International Agency for Research on Cancer (IARC) (2002). Fumonisin B1. Some Traditional Medicines, Some Mycotoxins, Naphthalene and Styrene.

[44] Rodrigues I., Handl J., Binder E. (2011). Mycotoxin occurrence in commodities, feeds and feed ingredients sourced in the Middle East and Africa. Food Addit. Contam. Part. B.

[45] Wilson T.M., Nelson P.E., Ryan T.B. (1985). Linking leukoencephalomalacia to commercial horse rations. Vet. Med..

[46] Jackson L., Nagan M., Olsen M. (2004). Fumonisins. Mycotoxins in Food: Detection and Control.

[47] Harrison L.R., Colvin B.M., Greene J.T., Newman L.E., Cole J.R. (1990). Pulmonary oedema and hydrothorax in swine produced by fumonisin B1, a toxic metabolite of *Fusarium moniliforme*. J. Vet. Diagn. Investig..

[48] Marasas W.F.O. (2001). Discovery and occurrence of the Fumonisins: A historical perspective. Environ. Health Perspect..

[49] Sultana N., Hanif N.Q. (2009). Mycotoxin contamination in cattle feed and feed ingredients. Pak. Vet. J..

[50] Pittet A. (1998). Natural occurrence of mycotoxins in foods and feeds: An updated review. Rev. Med. Vet..

[51] Zinedine A., Soriano J.M., Juan C., Mojemmi B., Moltó J.C., Bouklouze A., Cherrah Y., Idrissi L., El Aouad R., Mañes J. (2007). Incidence of ochratoxin A in rice and dried fruits from Rabat and Salé area, Morocco. Food Addit. Contam..

[52] European Food Safety Authority (EFSA) (2011). Scientific Opinion on the Risks for Public Health Related to the Presence of Zearalenone (ZEA) in Food. https://efsa.onlinelibrary.wiley.com/doi/pdf/10.2903/j.efsa.2011.2197.

[53] Mwanza M. (2007). An Investigation in South African Domesticated Animals, Their Products and Related Health Issues with Reference to Mycotoxins and Fungi. Master’s Thesis.

[54] Makun H.A., Dutton M.F., Njobeh P., Mwanza M., Kabiru A.Y. (2011). Natural multi-occurrence of mycotoxins in rice from Niger State, Nigeria. Mycotoxin Res..

[55] Bailly J., Guerre P., Nollet L.M.L., Toldrá F. (2016). Mycotoxin analysis in poultry and processed meats. Safety Analysis of Foods of Animal Origin.

[56] Ahamed S., Forester J.S., Bukovsky A., Winalasena J. (2001). Signal transduction through the rass/Erk pathway is essential for the mycoestrogen zearalenone-induced cell-cycle progression in MCF-7 cells. Mol. Carcinog..

[57] Krska R. How does climate change impact on the occurrence and the determination of natural toxins. Proceedings of the 7th International Symposium on Recent Advances in Food Analysis.

[58] Vaclavikova M., Malachova A., Veprikova Z., Dzuman Z., Zachariasova M., Hajslova J. (2013). Emerging’ mycotoxins in cereals processing chains: Changes of enniatins during beer and bread making. Food Chem..

[59] Jestoi M. (2008). Emerging Fusarium—mycotoxins fusaproliferin, beauvericin, enniatins, and moniliformin—a review. Crit. Rev. Food Sci. Nutr..

[60] Sanhueza C.E.P., Degrossi M.C. (2004). Moniliformin, a Fusarium mycotoxin. Soc. Mex. Micol. Xalapa.

[61] Zain M.E. (2011). Impact of mycotoxins on humans and animals. J. Saudi Chem. Soc..

[62] Thiel P.G., Meyer C.J., Marasas W.F.O. (1982). Natural occurence of moniliformin together with deoxynivalenol and zearalenone in Transkei corn. J. Agric. Food Chem..

[63] Bottalico A. (1998). Fusarium diseases of cereals: Species complex and related mycotoxin profiles in Europe. Eur. J. Plant. Pathol..

[64] Prosperini A., Berrada H., Ruiz M.J., Caloni F., Coccini T., Spicer L.J., Perego M.C., Lafranconi A. (2017). A review of the mycotoxin enniatin B. Front. Public Health.

[65] (2016). Biomin.net. https://www.biomin.net/.../emerging-mycotoxins-overview-and-occurrence.

[66] Jajić I., Dudaš T., Krstović S., Krska R., Sulyok M., Bagi F., Savić Z., Guljaš D., Stankov A. (2019). Emerging Fusarium mycotoxins fusaproliferin, beauvericin, enniatins, and moniliformin in serbian maize. Toxins.

[67] Marasas W.F.O., Kellerman T.S., Gelderblom W.C., Coetzer J.A., Thiel P.G., van der Lugt J.J. (1988). Leukoencephalomalacia in a horse induced by fumonisin B1 isolated from *Fusarium moniliforme*. J. Vet. Res..

[68] Chu F.S., Li G.Y. (1994). Simultaneous occurrence of fumonisin B1 and other mycotoxins in moldy corn collected from the People’s Republic of China in regions with high incidence of esophageal cancer. Appl. Environ. Microbiol..

[69] Soriano J.M., Dragacci S. (2004). Occurrence of Fumonisins in foods. Food Res. Int..

[70] Prelusky D.B., Rotter B.A., Rotter R.G. (1994). Mycotoxins in Grain.

[71] Li M., Harkema J.R., Islam Z., Cuff C.F., Pestka J.J. (2006). T-2 toxin impairs murine immune response to respiratory reovirus and exacerbates viral bronchiolitis. Toxicol. Appl. Pharmacol..

[72] Hymery N., Léon K., Carpentier F.G., Jung J.L., Parent-Massin D. (2009). T-2 toxin inhibits the differentiation of human monocytes into dendritic cells and macrophages. Toxicol. In Vitro.

[73] Kubosaki A., Aihara M., Park B.J., Sugiura Y., Shibutani M., Hirose M., Suzuki Y., Takatori K., Sugita-Konishi Y. (2008). Immunotoxicity of nivalenol after subchronic dietary exposure to rats. Food Chem. Toxicol..

[74] Bony S., Olivier-Loiseau L., Carcelen M., Devaux A. (2007). Genotoxic potential associated with low levels of the Fusarium mycotoxins nivalenol and fusarenon X in a human intestinal cell line. Toxicol. In Vitro.

[75] Miller J.D., Trenholm H.L. (1994). Mycotoxins in Grain: Compounds Other Than Aflatoxin.

[76] D’Mello J.P.F., Macdonald A.M.C. (1997). Mycotoxins. Anim. Feed Sci. Technol..

[77] Peltonen K., Jestoi M., Eriksen G.S. (2010). Health effects of moniliformin a poorly understood *Fusarium* mycotoxin. World Mycotoxin J..

[78] Zhang A., Cao J.-L., Yang B., Chen J.-H., Zhang Z.-T., Li S.-Y., Fu Q., Hugnes C.E., Caterson B. (2010). Effects of moniliformin and selenium on human articular cartilage metabolism and their potential relationships to the pathogenesis of Kashin-Beck disease. J. Zhejiang Univ. Sci. B.

[79] Juan-García A., Manyes L., Ruiz M.-J., Font G. (2013). Involvement of enniatins-induced cytotoxicity in human HepG2 cells. Toxicol. Lett..

[80] Prosperini A., Font G., Ruiz M.J. (2014). Interaction effects of *Fusarium enniatins* (A, A1, B and B1) combinations on in vitro cytotoxicity of Caco-2 cells. Toxicol. In Vitro.

[81] Young J.C., Fulcher R., Hayhoe J., Scott P., Dexter J. (1984). Effect of milling and baking on deoxynivalenol (vomitoxin) content of eastern Canadian wheats. J. Agric. Food Chem..

[82] Savard M.E. (1991). Deoxynivalenol fatty acid and glucoside conjugates. J. Agric. Food Chem..

[83] Sewald N., von Gleissenthall J.L., Schuster M., Müller G., Aplin R.T. (1992). Structure elucidation of a plant metabolite of 4-desoxynivalenol. Tetrahedron Asymmetry.

[84] Kamimura H. (1986). Conversion of zearalenone to zearalenone glycoside by *Rhizopus* sp.. Appl. Environ. Microbiol..

[85] Engelhardt G., Zill G., Wohner B. (1988). Transformation of the Fusarium mycotoxin zearalenone in maize cell suspension cultures. Naturwissenschaften.

[86] Mirocha C. (1981). Distribution and metabolism of zearalenone in a lactating cow. J. Am. Oil Chem. Soc..

[87] Plasencia J., Mirocha C.J. (1991). Isolation and characterization of zearalenone sulphate produced by *Fusarium* spp.. Appl. Environ. Microbiol..

[88] Broekaert N., Devreese M., de Baere S., de Backer P., Croubels S. (2015). Modified Fusarium mycotoxins unmasked: From occurrence in cereals to animal and human excretion. Food Chem. Toxicol..

[89] Coleman J., Blake-Kalff M., Davies T.G.E. (1997). Detoxification of xenobiotics by plants: Chemical modification and vacuolar compartmentation. Trends Plant. Sci..

[90] Conn E., Key J.L., Kosuge T. (1985). Chemical conjugation and compartmentalization: Plant adaptations to toxic natural products. Cellular and Molecular Biology of Plant Stress.

[91] Sandermann J. (1992). Plant metabolism of xenobiotics. Trends Biochem. Sci..

[92] Engelhardt G., Ruhland M., Wallnöfer P. (1999). Metabolism of mycotoxins in plants. Adv. Food Sci..

[93] Bryła M., Ksieniewicz-Woźniak E., Waśkiewicz A., Szymczyk K., Jędrzejczak R. (2018). Natural occurrence of nivalenol, deoxynivalenol, and deoxynivalenol-3-glucoside in Polish winter wheat. Toxins.

[94] Di Mavungu J.D., de Saeger S. (2011). Masked mycotoxins in food and feed: Challenges and analytical approaches. Determining Mycotoxins and Mycotoxigenic Fungi in Food and Feed.

[95] Berthiller F., Crews C., Dall’Asta C., de Saeger S., Haesaert G., Karlovsky P., Oswald I.P., Seefelder W., Speijers G., Stroka J. (2012). Masked mycotoxins: A review. Mol. Nutr. Food Res..

[96] Karlovsky P. (1999). Biological detoxification of fungal toxins and its use in plant breeding, feed and food production. Nat. Toxins.

[97] Berthiller F., Dall’Asta C., Schuhmacher R., Lemmens M., Adam G., Krska R. (2005). Masked mycotoxins: Determination of a deoxynivalenolglucoside in artificially and naturally contaminated wheat by liquid chromatography—Tandem mass spectrometry. J. Agric. Food Chem..

[98] Nakagawa H., Ohmichi K., Sakamoto S., Sago Y., Kushiro M., Nagashima H., Yoshida M., Nakajima T. (2011). Detection of new Fusarium masked mycotoxins in wheat grain by high-resolution LC–Orbitrap MS. Food Addit. Contam..

[99] Berthiller F. (2016). Unravelling the real threats of masked mycotoxins. https://www.allaboutfeed.net/Mycotoxins/Articles/2016/7/Unravelling-the-real-threats-of-masked-mycotoxins-2841508W/.

[100] Malachova A., Dzuman Z., Veprikova Z., Vaclavikova M., Zachariasova M., Hajslova J. (2011). Deoxynivalenol, deoxynivalenol-3-glucoside, and enniatins: The major mycotoxins found in cereal-based products on the Czech market. J. Agric. Food Chem..

[101] Dall’Asta C., Galaverna G., Mangia M., Sforza S., Dossena A., Marchelli R. (2009). Free and bound fumonisins in gluten-free food products. Mol. Nutr. Food Res..

[102] Galaverna G., Dall’Asta C., Mangia M., Dossena A., Marchelli R. (2009). Masked mycotoxins: An emerging issue for food safety. Czech. J. Food Sci..

[103] Humpf H.-U., Voss K.A. (2004). Effects of thermal food processing on the chemical structure and toxicity of fumonisin mycotoxins. Mol. Nutr. Food Res..

[104] Howard P.C., Churchwell M.I., Couch L.H., Marques M.M., Doerge D.R. (1998). Formation of *N*-(Carboxymethyl)fumonisin B_1_, following the reaction of fumonisin B_1_ with reducing sugars. J. Agric. Food Chem..

[105] Poling S.M., Plattner R.D., Weisleder D. (2002). *N*-(1-Deoxy-D-fructos-1-yl) fumonisin B_1_, the initial reaction product of fumonisin B_1_ and D-glucose. J. Agric. Food Chem..

[106] Seefelder W., Hartl M., Humpf H.U. (2001). Determination of *N*-(carboxymethyl) Fumonisin B_1_ in corn products by liquid chromatography/electrospray ionization—Mass spectrometry. J. Agric. Food Chem..

[107] Kim E.Y., Scott P.M., Lau B.P.-Y., Lewis D.A. (2002). Extraction of Fumonisins B_1_ and B_2_ from white rice flour and their stability in white rice flour, cornstarch, corn meal and glucose. J. Agric. Food Chem..

[108] Kim E., Scott P., Lau B. (2003). Hidden fumonisin in corn flakes. Food Addit. Contam..

[109] Park J.W., Scott P.M., Lau B.P.Y., Lewis D.A. (2004). Analysis of heat-processed corn foods for fumonisins and bound fumonisins. Food Addit. Contam..

[110] Seefelder W., Knecht A., Humpf H.U. (2003). Bound fumonisin-B_1_: Analysis of fumonisin-B_1_ glyco and amino acid conjugates by liquid chromatography–electrospray ionization–tandem mass spectrometry. J. Agric. Food Chem..

[111] Dall’Asta C., Galaverna G., Aureli G., Dossena A., Marchelli R. (2008). A LC/MS/MS method for the simultaneous quantification of free and masked fumonisins in maize and maize-based products. World Mycotoxin J..

[112] Kostelanska M., Dzuman Z., Malachova A., Capouchova I., Prokinova E., Skerikova A., Hajslova J. (2011). Effects of milling and baking technologies on levels of deoxynivalenol and its masked form deoxynivalenol-3-glucoside. J. Agric. Food Chem..

[113] Schollenberger M., Müller H.-M., Rüfle M., Suchy S., Drochner W. (2008). Redistribution of 16 fusarium toxins during commercial dry milling of maize. Cereal Chem. J..

[114] Lancova K., Hajslova J., Kostelanska M., Kohoutkova J., Nedelnik J., Moravcova H., Vanova M. (2008). Fate of trichothecene mycotoxins during the processing: Milling and baking. Food Addit. Contam. Part. A Chem. Anal. Control. Expo. Risk Assess.

[115] De Angelis E., Monaci L., Visconti A. (2014). Investigation on the stability of deoxynivalenol and DON-3 glucoside during gastro-duodenal in vitro digestion of a naturally contaminated bread model food. Food Control..

[116] Abid-Essefi S., Bouaziz C., Golli-Bennour E.E., Ouanes Z., Bacha H. (2009). Comparative study of toxic effects of zearalenone and its two major metabolites α-zearalenol and β-zearalenol on cultured human Caco-2 cells. J. Biochem. Mol. Toxicol..

[117] Scientific Committee on Food (SCF) (2002). Opinion of the Scientific Committee on Food on Fusarium Toxins. Part 6: Group Evaluation of T-2 Toxin, HT-2 Toxin, Nivalenol and Deoxynivalenol, Adopted on 26 February 2002. http://ec.europa.eu/food/fs/sc/scf/out123_en.pdf.

[118] Alassane-Kpembi I., Puel O., Oswald I.P. (2015). Toxicological interactions between the mycotoxins deoxynivalenol, nivalenol and their acetylated derivatives in intestinal epithelial cells. Arch. Toxicol..

[119] Kadota T., Furusawa H., Hirano S., Tajima O., Kamata Y., Sugita-Konishi Y. (2013). Comparative study of deoxynivalenol, 3-acetyldeoxynivalenol, and 15- acetyldeoxynivalenol on intestinal transport and IL-8 secretion in the human cell line Caco-2. Toxicol. In Vitro.

[120] (2015). Biomin.net. https://www.biomin.net/en/articles/can-low-levels-of-mycotoxins-harm-the-poultry-industry/.

[121] Chilaka C.A., De Boevre M., Atanda O.O., De Saeger S. (2017). The Status of *Fusarium* Mycotoxins in Sub-Saharan Africa: A Review of Emerging Trends and Post-Harvest Mitigation Strategies towards Food Control. Toxins.

[122] Ekwomadu T.I., Dada T.A., Akinola S.A., Nleya N., Mwanza M. (2021). Analysis of selected mycotoxins in maize from north-west South Africa using high performance liquid chromatography (HPLC) and other analytical techniques. Separations.

[123] Fandohan P., Gnonlonfin B., Hell K., Marasas W., Wingfield M. (2005). Natural occurrence of Fusarium and subsequent fumonisin contamination in preharvest and stored maize in Benin, West Africa. Int. J. Food Microbiol..

[124] Basappa S.C. (2009). Aflatoxins Formation, Analysis and Control.

[125] Van der Westhuizen L., Shephard G., Rheeder J.P., Burger H.-M., Gelderblom W., Wild C., Gong Y.Y. (2011). Optimising sorting and washing of home-grown maize to reduce fumonisin contamination under laboratory-controlled conditions. Food Control..

[126] Matumba L., van Poucke C., Ediage E.N., Jacobs B., de Saeger S. (2015). Effectiveness of hand sorting, flotation/washing, dehulling and combinations thereof on the decontamination of mycotoxin-contaminated white maize. Food Addit. Contam. Part. A.

[127] Wagacha J., Muthomi J. (2008). Mycotoxin problem in Africa: Current status, implications to food safety and health and possible management strategies. Int. J. Food Microbiol..

[128] Turner N., Subrahmanyam S., Piletsky S. (2009). Analytical methods for determination of mycotoxins: A review. Anal. Chim. Acta.

[129] Ryu D., Hanna M.A., Bullerman L.B. (1999). Stability of zearalenone during extrusion of corn grits. J. Food Prot..

[130] Desjardins A.E., Manandhar G., Plattner R.D., Maragos C.M., Shrestha K., McCormick S.P. (2000). Occurrence of Fusarium species and mycotoxins in Nepalese maize and wheat and the effect of traditional processing methods on mycotoxin levels. J. Agric. Food Chem..

[131] Yiannikouris A., Jouany J.-P. (2002). Mycotoxins in feeds and their fate in animals: A review. Anim. Res..

[132] Dellafiora L., Ruotolo R., Perotti A., Cirlini M., Galaverna G., Cozzini P., Buschini A., Dall’Asta C. (2017). Molecular insights on xenoestrogenic potential of zearalenone-14-glucoside through a mixed in vitro/in silico approach. Food Chem. Toxicol..

